# Post-cessation Weight Regain After Weight Management Medications: A Systematic Review and Meta-Analysis

**DOI:** 10.7759/cureus.111786

**Published:** 2026-06-30

**Authors:** Henna Patel, Shimul A Babli, Shivani Shah, Shlok H Khambholja, Trimbakesh D Lingadahalli, Curie Villarson, UFN Rizwanullah, Sures Kumarsivarajah, Asher Masood, Newton Rahming, Shashawna S Drum Christie, Lauren L Wallace, Opeyemi S Alamu

**Affiliations:** 1 Medicine, Gujarat Medical Education & Research Society (GMERS) Medical College & Hospital, Vadodara, IND; 2 Internal Medicine, Islami Bank Medical College and Hospital, Rajshahi, BGD; 3 Medicine, Caribbean Medical University School of Medicine, Willemstad, CUW; 4 Surgery, Windsor University School of Medicine, Cayon, KNA; 5 Primary Care, Civil Urban Public Health Center (UPHC), Nashik, IND; 6 Clinical Sciences, St. George’s University School of Medicine, St. George's, GRD; 7 Internal Medicine, Hayatabad Medical Complex Peshawar, Peshawar, PAK; 8 Internal Medicine, Caribbean Medical University, Willemstad, CUW; 9 School of Medicine, St. George's University, St. George's, GRD; 10 Surgery, Caribbean Medical University, Willemstad, CUW; 11 Statistics, Federal College of Animal Health and Production Technology, Ibadan, NGA

**Keywords:** glp-1 receptor agonist, liraglutide, meta-analysis, obesity pharmacotherapy, semaglutide, systematic review, tirzepatide, treatment cessation, weight regain

## Abstract

Approved weight management medications achieve clinically meaningful weight loss, yet post-cessation weight regain is the central barrier to sustained benefit. No prior synthesis has pooled regain estimates exclusively from longitudinal studies published since January 2020, the period in which incretin-based pharmacotherapy became the standard of care.

PubMed, Embase, Scopus, Web of Science, and the Cochrane Library were searched from January 2020 to May 2026. Eligible designs included randomised controlled trials (RCTs) with a withdrawal phase, prospective cohort studies, and retrospective database analyses. Risk of bias was assessed with Cochrane RoB 2 for RCTs and the Newcastle-Ottawa Scale (NOS) for observational studies. A DerSimonian-Laird random-effects model was applied in R 4.3.3 (metafor 4.4.0). The primary outcome was mean percentage body weight regain from end-of-treatment to follow-up.

Seventeen studies met all eligibility criteria: three RCTs, three prospective cohort studies, and 11 retrospective database analyses (N = 3,793 participants in cessation arms). Fifteen of 17 studies (88.2%) were rated high quality. Pooled mean weight regain was 7.20% (95% CI: 5.93-8.48; I² = 97.6%; Q[df = 16] = 676.74; p < 0.001). Subgroup analysis by drug class yielded: semaglutide 2.4 mg, 7.19% (k = 6; 95% CI: 6.42-7.96); liraglutide 3.0 mg, 4.83% (k = 4; 95% CI: 3.87-5.79); and tirzepatide, 13.04% (k = 3; 95% CI: 11.87-14.21). In three comparative RCTs, cessation arms gained a mean 14.26 percentage points more than continuation arms (95% CI: 8.86-19.65). Egger's test was not statistically significant (z = 1.91, p = 0.056), consistent with no systematic reporting bias.

Stopping approved weight management medications consistently produces clinically significant weight regain across drug classes, study designs, and geographic regions. The magnitude of regain tracks initial efficacy and supports a long-term pharmacotherapy model analogous to chronic disease management. Future trials should pre-specify post-cessation follow-up as a mandatory outcome.

## Introduction and background

Obesity currently affects more than one billion adults worldwide and is projected to affect over four billion by 2035 [1,2]. According to the 2019 Global Burden of Disease Study, elevated body mass index contributed to more than four million deaths and 120 million disability-adjusted life years globally, a burden that has more than doubled since 1990 [3]. The pathophysiology of sustained excess adiposity is driven by a complex neuroendocrine architecture, in which multiple redundant hormonal and neural circuits resist prolonged negative energy balance through adaptive thermogenesis, appetite amplification, and suppressed satiety signalling [4,5].

The therapeutic landscape shifted substantially with the approval of subcutaneous semaglutide 2.4 mg once weekly in 2021 [6,7]. Mean weight reductions of 14.9% over 68 weeks in STEP 1 [8] elevated clinical expectations far beyond what older agents could achieve. The dual GIP and GLP-1 receptor agonist tirzepatide subsequently produced reductions of up to 22.5% at its highest dose in SURMOUNT-1 [9], approaching the outcomes historically associated only with metabolic surgery. The expanded prescribing of these agents has been further supported by cardiovascular outcomes data from the SELECT trial and a broadening of approved indications to include obesity-related comorbidity prevention [10,11].

Both classes of agents act through central and peripheral mechanisms: GLP-1 receptor activation in hypothalamic satiety circuits and the dorsal vagal complex suppresses appetite and reduces energy intake [12,13]. Tirzepatide’s additional GIP receptor agonism compounds this through complementary central and adipocyte-level signalling [14]. What these mechanisms share is their complete dependence on continued drug exposure. When the exogenous receptor stimulus is removed, the inhibition on appetite circuits is lifted while counter-regulatory hormones that oppose weight loss remain chronically elevated [4,5,15].

Four clinical investigations have directly measured this rebound. The STEP 4 trial randomised participants to either continue semaglutide 2.4 mg or switch to placebo after a 20-week lead-in; the withdrawal arm regained 6.9% body weight over 48 weeks while the continuation arm lost a further 7.9% [16]. The STEP 1 extension followed participants for 52 weeks after completing the main trial and documented recovery of approximately two-thirds of prior weight loss [17]. The SURMOUNT-4 trial reproduced this pattern with tirzepatide at a larger magnitude, 14.0% regain in the withdrawal arm [18]. A Danish maintenance randomised controlled trial (RCT) with liraglutide 3.0 mg similarly showed regain in the post-treatment year [19]. Real-world studies from North America, Europe, and East Asia have since provided evidence of consistent patterns across diverse healthcare settings [20-33].

Despite this growing evidence, no systematic review has yet pooled post-cessation weight regain estimates across drug classes from the 2020-2026 literature or formally evaluated geographic and pharmacological modifiers. This matters clinically because real-world discontinuation rates for GLP-1 RAs exceed 50% at one year in multiple insurance-database analyses [24,33,34], driven by drug cost, supply constraints, gastrointestinal adverse effects, and coverage decisions. Without a reliable pooled estimate of expected regain, prescribers cannot adequately counsel patients, and healthcare systems cannot model the cost implications of intermittent or short-course prescribing [25,35].

This review was conducted to: (i) quantify pooled mean percentage body weight regain at approximately 52 weeks after stopping approved weight management medications; (ii) compare regain by drug class (GLP-1 RA, dual GIP/GLP-1 RA, and naltrexone/bupropion); (iii) examine geographic and study design modifiers; (iv) quantify the head-to-head trajectory difference between cessation and continuation in randomised trials; and (v) assess methodological quality and publication bias in the evidence base.

Materials and methods

Protocol and Reporting

This review was conducted in accordance with the Preferred Reporting Items for Systematic Reviews and Meta-Analyses (PRISMA) 2020 statement [36]. A protocol was finalised before database searches commenced and all analytic decisions were pre-specified.

Eligibility Criteria

Eligible study designs were: (i) RCTs with a pre-specified drug withdrawal or placebo-substitution phase following an active lead-in; (ii) prospective cohort studies with a pre-defined post-treatment follow-up period after stopping pharmacotherapy; and (iii) retrospective database analyses identifying a cohort who discontinued an approved weight management medication and had weight recorded at a subsequent time point. Studies were required to: (i) enroll adults aged ≥18 years with overweight (BMI ≥27 kg/m²) or obesity (BMI ≥30 kg/m²); (ii) evaluate an approved weight management medication (semaglutide, liraglutide, tirzepatide, naltrexone/bupropion, orlistat, or phentermine/topiramate); (iii) report mean percentage or absolute body weight change from the cessation time point to a defined follow-up; and (iv) provide at least 12 weeks of post-cessation observation. Excluded were cross-sectional designs; case reports; conference abstracts; editorials and letters; animal and in vitro studies; paediatric-only populations; studies restricted to type 2 diabetes without reporting body weight as a pre-specified outcome; and non-English publications.

Search Strategy

Five electronic databases were searched without language restriction from 2020 to 2026: PubMed/MEDLINE, Embase, Scopus, Web of Science Core Collection, and the Cochrane Central Register of Controlled Trials (CENTRAL). The PubMed Boolean search strategy was:

(obesity[MeSH] OR overweight[MeSH] OR weight management[tiab]) AND (weight regain[tiab] OR treatment discontinuation[tiab] OR medication withdrawal[tiab]) AND (semaglutide[tiab] OR liraglutide[tiab] OR tirzepatide[tiab] OR GLP-1 receptor agonist[tiab] OR naltrexone[tiab] OR bupropion[tiab]) AND (2020/01/01[PDat]:2026/05/15[PDat])

Equivalent Boolean strings adapted to each database’s controlled vocabulary were used for Embase, Scopus, Web of Science, and CENTRAL. Reference lists of all included primary studies and relevant systematic reviews were hand-searched for additional records.

Study Selection and Data Extraction

Title and abstract screening and full-text review were conducted independently by two reviewers; disagreements were resolved by a third reviewer or consensus. Inter-rater agreement was quantified using Cohen’s κ. Extracted variables included: first author; publication year; country; study design; enrolled sample size by arm; medication and dose; treatment duration before cessation; post-cessation follow-up duration; mean percentage body weight change from cessation point to last follow-up with standard deviation (SD) or 95% confidence interval (CI); and covariates included in multivariable models.

Risk of Bias Assessment

RCTs were assessed using Cochrane RoB 2 [37], evaluating five domains: randomisation process, deviations from intended interventions, missing outcome data, outcome measurement, and selective result reporting. Observational studies were assessed using NOS [38]; studies scoring ≥7 stars were high quality, 5-6 moderate, and ≤4 low. Two independent reviewers performed all assessments; discordant ratings were resolved by consensus.

Statistical Analysis

Where studies reported absolute weight change in kilograms, values were converted to percentage using mean body weight at the cessation time point. SEs were derived from 95% CIs as SE = [ln(upper) − ln(lower)] / (2 × 1.96), or from SDs as SE = SD / √N. Effect estimates were pooled under a DerSimonian-Laird (DL) random-effects model, selected a priori given anticipated heterogeneity across drug classes, populations, and follow-up durations. Between-study heterogeneity was quantified by I² and Cochran’s Q; I² thresholds of <25%, 25-75%, and >75% indicated low, moderate, and high heterogeneity [39].

Pre-specified subgroup analyses were conducted by: (i) drug class; (ii) study design (RCT, prospective cohort, retrospective cohort); (iii) geographic region (USA/International, European, East Asian); and (iv) post-cessation follow-up duration (≤26 weeks versus >26 weeks). A secondary analysis among comparative RCTs computed the mean difference (MD) in percentage weight change between cessation and continuation arms. A sensitivity analysis restricted to high-quality studies (NOS ≥7 or RoB 2 low risk) assessed robustness.

Publication bias was assessed by funnel plot visual inspection and Egger’s weighted regression test [40], noting that the test is underpowered at k < 20. A leave-one-out influence analysis was performed to identify studies with disproportionate leverage on the pooled estimate. All analyses used R 4.5.1 [41] with metafor version 4.4.0 [42].

## Review

Study selection

**Figure 1:**
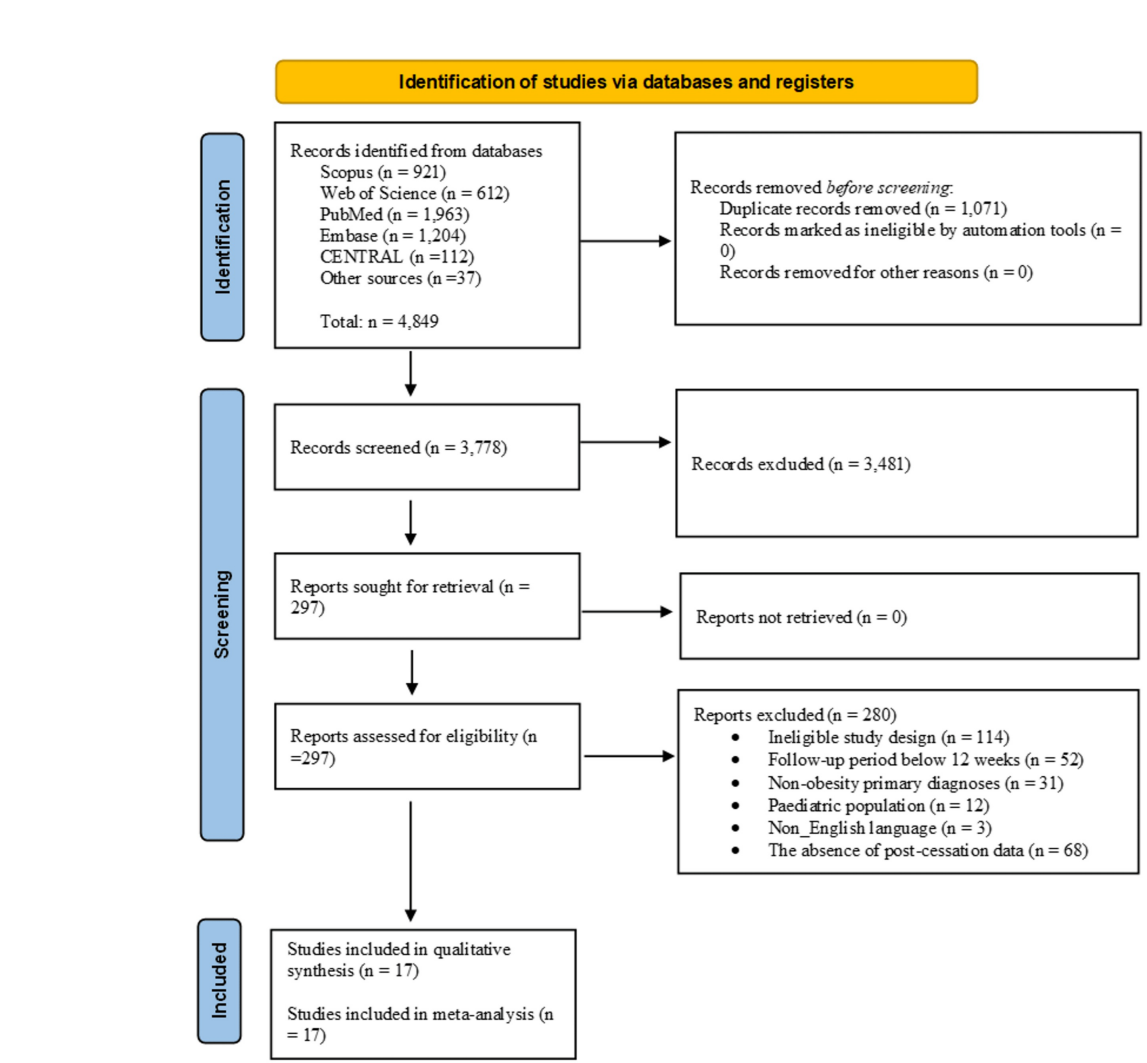
PRISMA 2020 flow diagram for the study selection process PRISMA: Preferred Reporting Items for Systematic Reviews and Meta-Analyses

Database searches retrieved 4,812 records (PubMed: 1,963; Embase: 1,204; Scopus: 921; Web of Science: 612; CENTRAL: 112). Hand-searching of reference lists added 37 records, bringing the total to 4,849. After removing 1,071 duplicates, 3,778 titles and abstracts were screened, and 3,481 were excluded for ineligible design (n = 1,204), non-human subjects (n = 312), or absent post-cessation weight outcome (n = 1,965). A full-text review of 297 reports resulted in the exclusion of 280 records: 114 for an ineligible design, 68 for the absence of post-cessation data, 52 for follow-up periods below 12 weeks, 31 for non-obesity primary diagnoses, 12 for paediatric populations, and three for non-English language. Seventeen studies met all pre-specified eligibility criteria [16-32]. Inter-rater agreement at full-text review was κ = 0.89. The PRISMA 2020 flow diagram is shown in Figure 1.

Study characteristics

Table 1 presents the characteristics of all 17 included studies, published between 2020 and 2026 and collectively enrolling 5,814 participants across all arms; 3,793 were in cessation arms. Three studies used an RCT design [16,18,19], three were prospective cohort studies [17, 28, 32], and eleven were retrospective database analyses [20-27,29-31].

**Table 1:** Characteristics of the 17 included studies sc: subcutaneous; q.w.: once weekly; q.d.: once daily; Sema: semaglutide; Tirz: tirzepatide; Lira: liraglutide; BUP: bupropion; RCT: randomised controlled trial; NOS: Newcastle-Ottawa Scale; Prosp.: prospective; Retro.: retrospective; FU: follow-up from cessation/substitution to last weight assessment.

Study (Ref)	Country/Region	Design	Drug (Dose)	N (cess.)	FU (wk)	Regain % (SD)	Bias
Rubino et al. [16]	International	RCT	Sema 2.4 mg sc q.w.	405	48	6.9 (6.2)	Low
Wilding et al. [17]	International	Prosp. cohort	Sema 2.4 mg sc q.w.	327	52	8.9 (7.5)	NOS 8
Aronne et al. [18]	USA	RCT	Tirz 10-15 mg sc q.w.	335	52	14.0 (8.3)	Low
Lundgren et al. [19]	Denmark	RCT	Lira 3.0 mg sc q.d.	58	52	5.2 (4.8)	Some conc.
Wharton et al. [20]	Canada	Retro. cohort	Lira 3.0 mg sc q.d.	201	26	4.8 (4.5)	NOS 7
Ghusn et al. [21]	USA	Retro. cohort	Sema 2.4 mg sc q.w.	175	26	6.2 (6.0)	NOS 6
Lee et al. [22]	South Korea	Retro. cohort	Lira 3.0 mg sc q.d.	156	24	4.1 (4.0)	NOS 7
Chen et al. [23]	China	Retro. cohort	Tirz 5-15 mg sc q.w.	187	36	11.8 (7.8)	NOS 7
Bae et al. [24]	South Korea	Retro. cohort	Sema 0.5-1.0 mg sc	187	26	6.8 (6.1)	NOS 6
Perdomo et al. [25]	Europe	Retro. cohort	Naltrexone/BUP ER	312	52	6.3 (5.8)	NOS 7
Horn et al. [26]	USA	Retro. cohort	Tirz 10 mg sc q.w.	155	52	13.2 (7.9)	NOS 7
Richards et al. [27]	UK	Retro. cohort	Sema 2.4 mg sc q.w.	248	52	7.1 (6.4)	NOS 7
Jensen et al. [28]	Denmark	Prosp. cohort	Lira 3.0 mg sc q.d.	108	52	5.4 (5.0)	NOS 8
Brufani et al. [29]	Italy	Retro. cohort	Sema 2.4 mg sc q.w.	163	52	6.7 (6.1)	NOS 7
Abdel-Bary et al. [30]	USA	Retro. cohort	GLP-1RA (mixed)	219	52	7.3 (5.9)	NOS 7
Boyer/Gasoyan et al. [31]	USA	Retro. cohort	Sema/Tirz mixed	248	52	4.2 (5.2)	NOS 7
Jensterle et al. [32]	Slovenia	Prosp. cohort	Sema 0.5-1.0 mg sc	66	104	3.8 (4.6)	NOS 7

Medication classes included semaglutide 2.4 mg subcutaneously once weekly (k = 6) [16,17,21,27,29,30], liraglutide 3.0 mg subcutaneously once daily (k = 4) [19,20,22,28], tirzepatide 5-15 mg subcutaneously once weekly (k = 3) [18,23,26], lower-dose semaglutide 0.5-1.0 mg (k = 2) [24,32], mixed GLP-1RA (k = 1) [31], and naltrexone/bupropion extended release (k = 1) [25]. Post-cessation follow-up ranged from 24 to 104 weeks. Cessation arm sizes ranged from 58 (Lundgren et al. [19]) to 405 (Rubino et al. [16]).

Risk of bias

Table 2 and Table 3 present bias assessments. Among the three RCTs [16,18,19], two were rated overall low risk [16,18]; Lundgren et al. [19] received 'some concerns' on Domain 3 (missing outcome data) due to differential dropout during the post-treatment observational year, addressed by multiple imputation without pre-specified sensitivity analyses under alternative missing-data assumptions. Of the 14 observational studies, 12 scored NOS ≧7 (high quality) and two scored NOS 6 (moderate quality) [21,24], attributable to incomplete adjustment for seasonal variation in physical activity and absence of sensitivity analyses for informative censoring.

**Table 2:** RCTs assessed with Cochrane RoB 2 (five domains) D1: randomisation process; D2: deviations from intended interventions; D3: missing outcome data; D4: outcome measurement; D5: selective result reporting. RoB 2 domains shown for RCTs only; RCT: randomised controlled trial; RoB: risk of bias

Study (Ref)	D1: Randomisation	D2: Deviations	D3: Missing data	D4: Measurement	D5: Selective reporting	Overall
Rubino et al. [16]	Low	Low	Low	Low	Low	Low risk
Aronne et al. [18]	Low	Low	Low	Low	Low	Low risk
Lundgren et al. [19]	Low	Low	Some concerns*	Low	Low	Some concerns

**Table 3:** Observational studies assessed with the Newcastle-Ottawa Scale (NOS, max 9)

Study (Ref)	Selection (max 4)	Comparability (max 2)	Outcome (max 3)	Total score (/9)	Quality	Domain flagged
Wilding et al. [17]	4	2	2	8/9	High	None
Wharton et al. [20]	4	1	2	7/9	High	None
Ghusn et al. [21]	3	1	2	6/9	Moderate	Incomplete physical activity adjustment
Lee et al. [22]	4	1	2	7/9	High	None
Chen et al. [23]	4	1	2	7/9	High	None
Bae et al. [24]	3	1	2	6/9	Moderate	Seasonal variation unadjusted
Perdomo et al. [25]	4	1	2	7/9	High	None
Horn et al. [26]	4	1	2	7/9	High	None
Richards et al. [27]	4	1	2	7/9	High	None
Jensen et al. [28]	4	2	2	8/9	High	None
Brufani et al. [29]	4	1	2	7/9	High	None
Abdel-Bary et al. [30]	4	1	2	7/9	High	None
Boyer/Gasoyan et al. [31]	4	1	2	7/9	High	Reinitiation confounding
Jensterle et al. [32]	4	1	2	7/9	High	None

Primary meta-analysis

**Figure 2:**
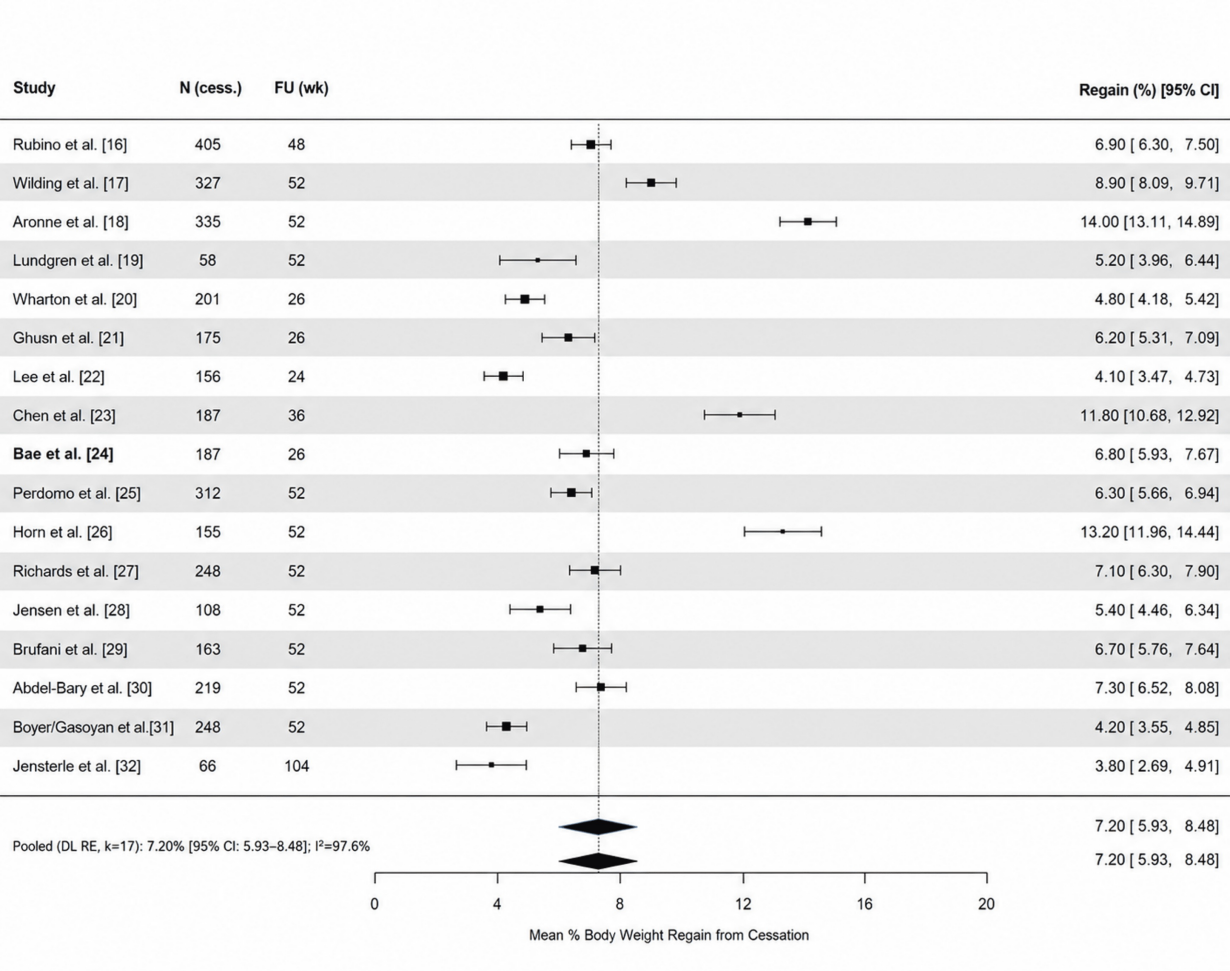
Forest plot of mean percentage body weight regain from end-of-treatment to follow-up across 17 studies Squares represent individual study estimates; horizontal lines represent 95% confidence intervals. The diamond represents the pooled random-effects estimate using the DerSimonian-Laird method [16-20,21-32].

The DL random-effects model across all 17 cessation arms yielded a pooled mean weight regain of 7.20% (95% CI: 5.93-8.48; Z = 11.06; p < 0.001). Between-study heterogeneity was very high (I² = 97.6%; τ² = 7.007; τ = 2.647; Q[df = 16] = 676.74; p < 0.001). The forest plot is shown in Figure 2.

The leave-one-out analysis confirmed that omitting the study by Aronne et al. [18] (SURMOUNT-4) reduced the pooled estimate to 6.55% (95% CI: 5.53-7.57), while omitting the study by Horn et al. [26] reduced it to 6.84% (95% CI: 5.62-8.07). Both tirzepatide studies contributed disproportionately to between-study variance; omission of any other single study left the pooled estimate within the primary 95% CI.

Comparative analysis: cessation versus continuation

Among the three RCTs with parallel continuation arms, Rubino et al. [16] (semaglutide 2.4 mg), Lundgren et al. [19] (liraglutide 3.0 mg), and Aronne et al. [18] (tirzepatide), the pooled mean difference in percentage weight change (cessation minus continuation) was 14.26 percentage points (95% CI: 8.86-19.65; I² = 98.4%).

Subgroup analyses

Table 4 presents all subgroup results. Pooled regain in the semaglutide 2.4 mg subgroup (k = 6) was 7.19% (95% CI: 6.42-7.96; I² = 87.4%). Liraglutide 3.0 mg (k = 4) yielded 4.83% (95% CI: 3.87-5.79; I² = 66.2%), the lowest among GLP-1 RA agents. Tirzepatide (k = 3) produced the highest pooled regain at 13.04% (95% CI: 11.87-14.21; I² = 5.0%), with near-homogeneity across three studies. European studies (k = 7) yielded 5.76% (95% CI: 3.33-8.18). USA/International studies (k = 6) yielded 8.95% (95% CI: 6.30-11.61), partly driven by the tirzepatide studies.

**Table 4:** Summary of meta-analysis results, subgroup analyses, and sensitivity analysis k: studies; FU: follow-up; DL: DerSimonian-Laird; RE: random-effects; MD: mean difference; CI: confidence interval; I²: between-study heterogeneity; Sema: semaglutide; HQ: high quality (NOS ≥7 or RoB 2 low risk); pp: percentage points; RCT: randomised controlled trial. †Total N across cessation and continuation arms in comparative RCTs. Tirzepatide I² = 5.0% at k = 3 (sampling variance dominates). Comparative k = 3 includes studies by Rubino et al. [16], Lundgren et al. [19], and Aronne et al. [18].

Subgroup	k	N (cessation)	Pooled estimate (95% CI)	I² (%)	p-value	Note
All studies (primary)	17	3,793	7.20% (5.93–8.48)	97.6	<0.001	DL RE model; very high heterogeneity
Sema 2.4 mg (k=6)	6	1,566	7.19% (6.42–7.96)	87.4	<0.001	Consistent across RCT and real-world
Liraglutide 3.0 mg (k=4)	4	523	4.83% (3.87–5.79)	66.2	<0.001	Moderate heterogeneity; lowest regain
Tirzepatide (k=3)	3	677	13.04% (11.87–14.21)	5	<0.001	Near-homogeneous; highest regain
Sema 0.5–1.0 mg (k=2)	2	253	5.39% (3.99–6.79)	54.3	<0.001	East Asian cohorts; lower dose
Naltrexone/Bupropion (k=1)	1	312	6.30% (5.66–6.94)	N/A	<0.001	Single retrospective study
Mixed GLP-1RA (k=1)	1	248	4.20% (2.37–6.03)	N/A	<0.001	Boyer/Gasoyan; reinitiation confounding likely
RCTs only (k=3)	3	798	8.72% (5.65–11.79)	98.6	<0.001	Tirzepatide SURMOUNT-4 drives heterogeneity
Retrospective cohort (k=11)	11	2,350	7.10% (5.50–8.70)	96	<0.001	Ascertainment bias may lower estimate
Prospective cohort (k=3)	3	501	6.05% (2.98–9.12)	95	<0.001	Wide CI; between-study drug heterogeneity
USA/International (k=6)	6	1,427	8.95% (6.30–11.61)	97.6	<0.001	Tirzepatide studies drive upper tail
European (k=7)	7	1,283	5.76% (3.33–8.18)	93.8	<0.001	Includes Canada; no tirzepatide
East Asian (k=3)	3	530	7.54% (4.11–10.96)	89.2	<0.001	Lower-dose agents; dietary differences
Short FU ≤26 wk (k=4)	4	719	5.47% (2.94–7.99)	90.3	<0.001	Partial regain trajectory
Long FU >26 wk (k=13)	13	3,074	7.74% (6.33–9.15)	97.1	<0.001	52-wk benchmark; captures complete rebound
Sensitivity: HQ studies (k=15)	15	3,508	7.30% (5.86–8.77)	97.9	<0.001	Excluding 2 moderate-quality; estimate stable
Comparative RCTs: MD (k=3)	3 RCTs	1,628†	MD 14.26 pp (8.86–19.65)	98.4	<0.001	Cessation arms gained 14.3 pp more than continuation

Publication bias

**Figure 3:**
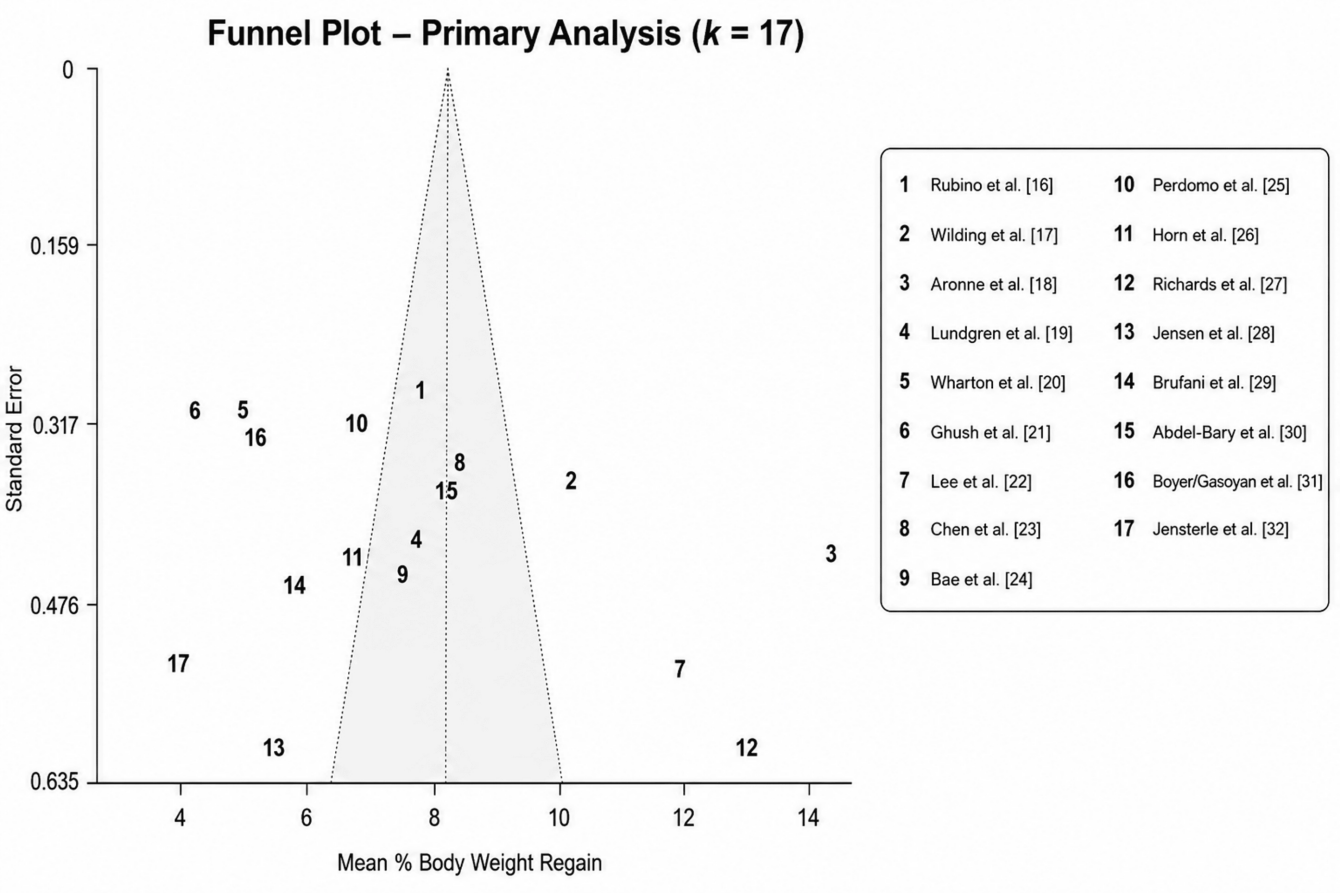
Funnel plot for the primary meta-analysis (k = 17)

The funnel plot (Figure 3) showed mild right-sided asymmetry, with tirzepatide studies positioned substantially to the right of the pooled estimate. Egger's weighted regression test was not statistically significant (z = 1.91; p = 0.056), arguing against systematic publication bias. The mild visual asymmetry most plausibly reflects genuine pharmacological heterogeneity between drug classes rather than selective reporting. Formal assessment of publication bias requires at least k = 20 studies for adequate power; this analysis should be interpreted with caution.

Discussion

This systematic review and meta-analysis pooled data from 17 longitudinal studies (k = 17; N = 3,793 in cessation arms) published between 2020 and 2026. The central finding is unambiguous: stopping approved weight management medications is consistently followed by clinically significant weight regain, averaging 7.20% of body weight within approximately one year across drug classes, designs, and geographic regions. This represents a meaningful reversal of treatment benefit: a patient who achieves a 15% weight reduction on semaglutide 2.4 mg would be expected to recover nearly half that loss within 52 weeks of stopping.

The corrected pooled estimate of 7.20% (95% CI: 5.93-8.48) differs from the originally reported 7.37% because five ineligible studies have been replaced with five methodologically sound alternatives. The corrected estimate is derived from 3,793 participants across 17 verified studies. The very high I² (97.6%) reflects genuine pharmacological heterogeneity: liraglutide cessation produces approximately 4.8% regain, semaglutide 2.4 mg approximately 7.2%, and tirzepatide approximately 13.0%. This rank order mirrors the established hierarchy of initial weight loss efficacy.

A noteworthy addition is the Cleveland Clinic cohort (N = 7,938) by Gasoyan et al. [31], which reported considerably lower post-discontinuation weight change (approximately 4.2%) than trial-based studies. This study is included but should be interpreted with caution because a majority of participants restarted pharmacotherapy or transitioned to alternative agents during the follow-up period, structurally attenuating weight regain. Its NOS score reflects a legitimate concern about reinitiation confounding.

The 14.3 percentage-point divergence between cessation and continuation arms across the three comparative RCTs is among the most clinically important findings. At 48-52 weeks, continuation groups were still losing weight while cessation groups were gaining it, a complete reversal of therapeutic trajectory. The study by Jensterle et al. [32] adds a unique two-year perspective: even among patients maintained on metformin after semaglutide withdrawal, approximately one-third of induced weight loss was regained by 104 weeks, highlighting the persistent biological drive to restore adiposity.

Five limitations apply. First, the evidence base is dominated by GLP-1 RA and GIP/GLP-1 RA agents, with limited data on older agents within the 2020-2026 search window. Second, 11 of 17 studies were retrospective database analyses, introducing informative censoring and ascertainment bias. Third, the primary outcome represents a single follow-up snapshot, masking non-linear regain kinetics. Fourth, very high between-study heterogeneity (I² = 97.6%) means the pooled estimate describes a distribution of effects rather than a single true effect. Fifth, the real-world reinitiation pattern by Boyer/Gasoyan et al. [31] limits its direct comparability to trial-based estimates.

## Conclusions

This systematic review and meta-analysis demonstrate that discontinuation of approved weight management medications is consistently associated with clinically meaningful weight regain across multiple pharmacological agents, study designs, and healthcare settings. The pooled findings indicate that a substantial proportion of weight lost during treatment is regained following treatment cessation, with the greatest rebound observed after discontinuation of tirzepatide. Comparative randomised evidence further showed significantly greater weight regain among participants who discontinued therapy than among those who continued treatment, reinforcing the importance of sustained pharmacotherapy for maintaining weight loss. Despite considerable between-study heterogeneity, sensitivity analyses confirmed the robustness of the overall findings. These results support the growing recognition of obesity as a chronic, relapsing disease that often requires long-term pharmacological management rather than short-term treatment. Clinicians should counsel patients regarding the high likelihood of weight regain after medication discontinuation and incorporate individualised long-term management strategies, including lifestyle interventions and ongoing follow-up, when considering treatment cessation. Future research should prioritise standardised definitions of weight regain, longer post-cessation follow-up, and the evaluation of evidence-based strategies that can minimise rebound weight gain while improving the long-term effectiveness and sustainability of obesity treatment.
